# Unveiling challenges in real-time PCR strategies for detecting treatment failure: observations from clinical trials on chronic Chagas disease

**DOI:** 10.3389/fpara.2023.1260224

**Published:** 2023-09-26

**Authors:** Alejandro G. Schijman

**Affiliations:** Laboratorio de Biología Molecular de la Enfermedad de Chagas, Instituto de Investigaciones en Ingeniería Genética y Biología Molecular Dr Héctor N. Torres (INGEBI), Consejo Nacional de Investigaciones Científicas y Técnicas (CONICET), Buenos Aires, Argentina

**Keywords:** polymerase chain reaction, Chagas disease, drug resistance, benznidazole, nifurtimox, dormancy, treatment failure, Trypanosoma cruzi

## Abstract

Chagas disease (CD) caused by Trypanosoma cruzi remains a Neglected Tropical Disease with limited access to diagnosis and treatment, particularly for chronically infected patients. Clinical trials are underway to improve treatment using new drugs or different regimens, and Real-Time PCR is used to assess the parasitological response as a surrogate biomarker. However, PCR-based strategies have limitations due to the complex nature of T. cruzi infection. The parasite exhibits asynchronous replication, different strains and clones, and diverse tissue tropism, making it challenging to determine optimal timeline points for monitoring treatment response. This mini-review explores factors that affect PCR-based monitoring and summarizes the endpoints used in clinical trials for detecting treatment failure. Serial sampling and cumulative PCR results may improve sensitivity in detecting parasitemia and treatment failure in these trials.

## Introduction

Chagas disease (CD), caused by the protozoan Trypanosoma cruzi, poses a significant public health threat in many regions worldwide (WHO, 2023). Transmission to humans primarily occurs through triatomine vectors, but other routes such as *in utero* transmission, contaminated meals, blood transfusions, or organ transplants are also possible. CD remains classified as a Neglected Tropical Disease, with limited access to efficient diagnosis and treatment, hampering efforts to control its impact on affected populations.

Current available therapies, such as benznidazole (BZN) and nifurtimox (NFX) have limitations in efficacy and safety, particularly in chronic adult CD patients (Moraes et al., 2014).

Clinical trials using different drug regimens and exploring new drugs in the pharmaceutical industry portfolio are underway to address this issue (Morillo et al., 2015; Morillo et al., 2017; Villar et al., 2019; Cafferata et al., 2020; Alonso-Vega et al., 2021; Torrico et al., 2021; Altcheh et al., 2023).

One of the challenges in assessing treatment efficacy is the reliance on molecular methods, particularly Real-Time PCR, to detect parasitic loads in peripheral blood (Schijman et al., 2011; Moreira et al., 2013; Ramírez et al., 2015). These methods have a limit of detection, and non-detectable findings cannot confirm parasitological eradication, leading to cure. Only detectable results can indicate treatment failure. This limitation hampers the ability to accurately determine the success of therapeutic interventions.

The lifecycle of *T. cruzi* further complicates the assessment of treatment effectiveness. Infection begins with motile extracellular trypomastigotes, which invade different cell types and convert into amastigotes, replicating in the host cytoplasm. Amastigotes then convert back to trypomastigotes, breaking the host cell membrane for parasite dissemination to the interstice and bloodstream. Recent studies have highlighted the asynchronous nature of intracellular parasite replication, the occurrence of dormancy and amastigotes’ growth plasticity to adapt and recover from sublethal drug exposure (Dumoulin and Burleigh, 2018; Sanchez-Valdez et al., 2018; Ward et al., 2020).

Another critical factor in treatment response is the genetic diversity and population structure of *T. cruzi*. CD patients may be infected with multiple parasite strains and clones, each with different tissue tropism (Macedo and Pena, 1998). The presence of parasites in the bloodstream may not necessarily reflect the ones responsible for clinical manifestations and drug susceptibilities. Different subpopulations of the parasite may be responsible for variations in treatment responses observed in multicenter trials.

Moreover, certain life-cycle stages may be less sensitive to treatment, and the ability of parasites to reside in metabolically distinct tissue compartments can significantly affect drug susceptibility (Barrett et al., 2019). These complexities highlight the need for more comprehensive approaches when evaluating treatment efficacy and drug response in CD.

## Parasite life-cycle stages and drug susceptibility

Benznidazole and nifurtimox exhibit pleiotropic properties with multiple effects on the target organism. This complexity can give rise to resistance in various ways. It is important to note that *T.cruzi* resistance observed in laboratory settings where *in vitro* resistance is generated may differ significantly from the resistance patterns observed in natural populations. This underscores the need of considering the interplay between laboratory studies and real-world observations when addressing drug resistance in *T.cruzi*.

The intracellular life cycle of *T. cruzi in vivo* is more intricate than previously assumed, revealing varying degrees of drug susceptibility among different parasite stages.

The research conducted by Moraes et al. (2014) highlights that trypomastigote forms have a higher capacity to withstand the trypanocidal effects of BZN and NFX compared to replicative epimastigotes and amastigotes. On the other hand, epimastigotes and amastigotes are more susceptible to a cumulative, trypanocidal, and trypanostatic impact of these drugs.

Revollo et al. (2019) investigated the susceptibility patterns of T. cruzi stages to BZN and NFX *in vitro* using a range of twenty-one T. cruzi strains from three different DTUs (Discrete Typing Units) isolated from patients, reservoirs, and triatomines across various geographical origins (Revollo et al., 1998). On the basis of the Epidemiological cut-off value (CO wt) (Kahlmeter, 2014), the authors computed the susceptibility threshold (COwt) of the T. cruzi life cycle forms against BZN and NFX from a panel of previously characterized strains and observed that the trypomastigote form exhibits higher tolerance to the toxic effects of both drugs compared to the other stages.

The study by Dumoulin and Burleigh (2018) revealed that intracellular amastigotes have the ability to adjust their proliferation rates as a survival strategy. They can range from minimal to rapid growth by accumulating within the G1 phase and then being released. The variation in doubling times differed between T.cruzi strains, suggesting that the metabolite levels or their thresholds, needed to initiate the S phase were also different in different strains. Treatment with BZN was found to inhibit amastigote proliferation in a concentration-dependent manner. Interestingly, when the drug was removed, the recovery of amastigotes was inversely proportional to the drug concentration used to treat them (Dumoulin and Burleigh, 2018).

## Strain diversity, histotropism and drug exposure

Studies have shown that *T. cruzi* strains isolated from triatomine vectors and vertebrate hosts exhibit multiclonality (Morel et al., 1986; Oliveira et al., 1998). During the interaction between the parasite and the human host in the chronic infection, certain subpopulations of T. cruzi may be selected and their differential tissue tropism can affect their distribution in the infected host (Vago et al., 2000). This selective role played by the human host can be exemplified by studies done in parasite isolates obtained from clinical samples of the first human CD case reported by Carlos Chagas: a two year-old girl named Berenice (Chagas, 1909). Indeed, two T. cruzi populations were isolated from Berenice Soares de Moura: one when she was a 55 years old- woman and another one when she reached 71 years of age (De Lana et al., 1996). The zymodeme and schizodeme profiles of these isolates diverged, which could be attributed to either a reinfection with a different strain or the presence of a heterogeneous population since the primary infection. Indeed, as T. cruzi is composed of heterogeneous populations, it is possible for the same host to be simultaneously infected by different strains. The clonal histotropic model suggests that the heterogeneity and multiclonality of a strain determine its differential tissue tropism, leading to variations in the clinical presentation of the disease (Macedo and Pena, 1998). It has been observed that clones found in patients’ cardiac tissues differed from those in the esophagus (Vago et al., 2000), and divergent subpopulations were identified in different tissue slices from the same heart explant in patients undergoing heart transplant (Burgos et al., 2010). Additionally, the occasional reactivation of parasites residing in target tissues may contribute to the dynamic behavior of T. cruzi infection, resulting in the appearance of new replication sites in different anatomical locations, as frequently observed in immunosuppressed patients with Chagas reactivation leading to panniculitis, myocarditis, or meningoencephalitis (Burgos et al., 2008; Burgos et al., 2010).

It has been reported that the epimastigote and amastigote forms of TcI strains are significantly less sensitive to BZN or NFX- mediated growth inhibitory effect compared to those belonging to TcII and TcV (Revollo et al., 2019). Strains CL (TcVI) and Colombiana (TcI), known to be susceptible and highly resistant, respectively, to BZN and NFX *in vivo* (Filardi and Brener, 1987), exhibit similar *in vitro* susceptibility in the amastigote stage (Canavaci et al., 2010).

*In vitro* experiments conducted by MacLean et al. (2018) demonstrated that strain PAH179 (TcV) exhibited marked resistance to posaconazole. This reduced sensitivity was attributed to the slow doubling and cycling time of this strain, which resulted in ergosterol biosynthesis inhibition by posaconazole only after multiple rounds of division. The lack of effect of posaconazole on the non-replicative trypomastigote form further supported this observation.

Characterization of transcriptomic profiles of BZN-resistant *T. cruzi* clones has revealed a wide array of genes from different metabolic pathways associated with the BZ-resistant phenotype. This indicates that parasite resistance mechanisms are multifactorial and complex (García-Huertas et al., 2017; Lima et al., 2023). Functional analysis has enabled the identification of relevant biological processes linked to the resistance phenotype, including changes in RNA processing and translation, antioxidant defence, as well as inter- and intracellular molecular transport (Lima et al., 2023).

Parasite genotyping can aid in understanding the differences in outcomes observed across different trials conducted in various geographical regions, as well as in detecting naturally resistant populations (Parrado et al., 2019; Muñoz-Calderón et al., 2023). Biorepositories containing well-conserved blood samples and extracted DNAs can be valuable for typing parasite populations from previous trials. The DNDi-CH-E1224-001 (Ramírez et al., 2022) and TESEO trials (Alonso-Vega et al., 2021) have incorporated DTU genotyping into their study designs.

## Dormancy and treatment failure

Sanchez-Valdez et al. (2018) made a ground breaking discovery by identifying drug-resistant persister subpopulations in T. cruzi. These dormant parasites have the unique characteristic of either not replicating or displaying slow replication rates. Moreover, they undergo metabolic changes, including reduced DNA synthesis and widespread downregulation of protein translation, in comparison to actively replicating cells (as shown in the studies by Van den Bergh et al., 2017, and Barrett et al., 2019).

There is a need to consider the potential role of dormancy mechanisms in the context of chronic infections. As the infection progresses to its chronic stage the parasite may encounter conditions that reduce its replication rate. For instance, certain types of DNA damage in *T.cruzi* such as double-strand breaks can trigger cell cycle arrest followed by repair without leading to parasite cell death. This repair process might inadvertently contribute to a dormancy-like state, where the parasite remains metabolically active but exhibits limited replication (Repolês et al, 2020).Dormant forms can arise spontaneously, independent of any drug pressure. Additionally, they possess a remarkable resilience to compounds that are highly effective against the actively replicating parasites.

The inability of trypanocidal drugs to completely eradicate replicative amastigote nests suggests the occurrence of transient dormancy within a specific subpopulation among the intracellular amastigote forms. These dormant cells can enter a state of quiescence during drug treatment and have the potential to resume replication once the treatment is stopped. Taylor and coworkers (2020) have hypothesized that the presence of such dormant nests may function as a form of intracellular “herd-protection,” where those parasite cells situated in an inner core of an amastigote nest would be less exposed to the drug compared to those amastigotes located at the periphery. As a result, the drug concentration within the host cell may be depleted before it can effectively target the core amastigotes, contributing to the survival and persistence of the dormant parasites. Such a phenomenon would pose challenges in completely eradicating *T. cruzi* infections and underscore the need for novel therapeutic approaches targeting these drug-resistant persister subpopulations.

*In vitro* studies using TUNEL assays and 5-ethynyl-2’-deoxyuridine (EdU) labeling have shown that replicating amastigotes, amastigote-to-trypomastigote differentiating forms and non-replicative trypomastigotes can coexist concurrently in a same host cell. The dynamics of parasite replication in each host cell does not follow a predictable or tightly regulated pattern, either *in vitro* or *in vivo*, at any phase of the disease or in specific infected tissues (Taylor et al., 2020).

Barrett et al. (2019) conducted a review of mechanisms used by protozoan parasite stages to enter a dormant state and establish persistent infections. To study the proliferation of T. cruzi in the colon of chronically infected mice, they incorporated EdU into DNA to track the replication status. By imaging infection foci at the single-cell level, they discovered that T. cruzi parasites were three times more likely to be in the S-phase during the acute stage of murine infection in the colon compared to the chronic stage. This finding suggested that a lower rate of parasite replication was associated with chronic colonic infections. Interestingly, the majority of infected murine cells did not survive T. cruzi infection for more than 14 days, indicating that persistence involved regular cycles of replication, host cell lysis, and reinfection (as mentioned in the study by Ward et al., 2020). This insight sheds light on the dynamics of T. cruzi infection and the potential mechanisms of persistence within the host.

## Clinical trials endpoints and measuring outcomes using Real Time PCR

Real-Time PCR remains the main laboratory tool for assessing treatment efficacy in clinical trials for Chagas disease. It is crucial to have well-standardized DNA extraction methods and commercially available Real-Time PCR kits, accompanied by internal and external quality controls (Ramírez et al., 2015; Ramírez et al., 2017) and reliable standard curves for quantification for expressing parasitic loads or target gene copies per unit of sample volume (Ramírez et al, 2015; Muñoz-Calderón et al., 2021). Quantitative PCR would permit establishing a minimal difference in parasitic loads between subsequent samples of a same patient under follow-up to interpret it as a true decrease or increase in parasitemia and accordingly treatment response improvement or failure.

In clinical practice, the concept of the “minimum clinically significant difference” (MCID) is often used to determine whether a change in a measured parameter, such as parasitic load, is meaningful from a clinical perspective. The MCID is the smallest change in a measurement that is considered to be clinically relevant and not just due to random variability. This depends on several factors, including the specific parasite population, DTU or strain being studied, the testing method used, and the clinical context. It is essential to consider the sensitivity and precision of the testing method, the variability in the measurement, and the natural course of the disease. However, this has not been done in the context of Chagas disease trials, yet.

Harmonization and agreement on criteria for using PCR in clinical trials for Chagas disease can be challenging due to various factors, including scientific, practical, and logistical considerations. Some debatable points that may arise during discussions and negotiations for harmonization are presented in **Table 1**.

**Table 1:** Debatable points regarding harmonization and agreement on criteria for using Polymerase Chain Reaction (PCR) in clinical trials for Chagas disease.

Issue	Key point of agreement	Points Needing Discussion
**PCR as surrogate marker of treatment response**	A positive PCR result after treatment is an effective marker of treatment failure.	Is there a clinical benefit in falling parasitic load?
**Inclusion Criteria**	There is no consensus regarding the need of a PCR positive result for enrollment of a patient in a trial	Inclusion criteria should be only to have a positive baseline PCR result. Should patients with positive serology but non detectable PCR results also be enrolled?
**Endpoint- measuring outcomes**	There is no consensus regarding the optimal timeline points to use PCR for monitoring treatment response	What is the recommended minimum number of timeline points to best measure efficacy among different studies?
**PCR related methodology**	In multicenter trials it is recommended to use a same standardized operative procedure including sample volume, sample collection, storage and transportation, DNA extraction method and Real Time PCR protocol or commercial kit	If similar PCR related reagents cannot be acquired in different sites participating in a multicenter trial, should a pilot study be done to harmonize techniques and enable comparison of results among the different PCR settings? Would be external quality control assurance a valid strategy for this purpose?
**PCR output**	A qualitative PCR positive result is valid enough to address treatment failure	Is it necessary to provide quantitative PCR results expressed as parasitic loads for monitoring treatment response in clinical trials? Should a “minimum clinically significant difference” (MCID) in parasitic loads be established for addressing improvement or failure of treatment?
**Geographical factors**	Treatment failure detection vary by geography, even within a same country.	Is this variability due to *T. cruzi* genetic diversity, including natural drug resistance and/or due to the immunological host background, and to which extent can this variability be attributed to PCR technical issues?

The minimum set of PCR timepoints required to detect treatment failure early needs to be agreed upon by the community involved in Chagas disease trials. Some trials have included a positive PCR result at baseline as an inclusion criterion for patients in a treatment arm. The clinical sensitivity of PCR in the chronic stage varies between 50% and 70% and is dependent on the geographical region. This has been observed by comparing PCR positivity and parasitic loads in baseline samples from patients enrolled in multicentric trials. For example, in the BENEFIT trial, treatment with benznidazole (BZN) was effective for patients from Argentina and Brazil (80% of the study population) but not for participants from Colombia and El Salvador (Morillo et al., 2015). This difference could be related to the distribution of parasite strains and DTUs (Discrete Typing Units), which can have different gene dosage for the molecular targets used in PCR. Furthermore, in certain regions, such as Minas Gerais, Brazil, primary resistant strains may prevail.

Serial sampling studies have been conducted in an attempt to increase the clinical sensitivity of PCR for detecting parasitic loads in pre-treated chronic Chagas disease patients (Parrado et al., 2019). In the DNDi-CH-E1224-001 trial, up to three samples were collected at each timepoint. Samples 1 and 2 were collected on the same day, with the former containing 10 ml and the second one containing 5 ml of blood in both cases mixed with one volume of the stabilizing agent. A third sample (10 ml) was collected seven days later if the PCR results from the previous two ones were non-detectable. The timing of the third sample collection was based on the assumption that T. cruzi intracellular replication generally lasts for 4-5 days, although it can be slower for certain isolates (Dumoulin and Burleigh, 2018).

Comparison of PCR positivity between individual samples did not show significant differences, indicating that collecting 5 ml or 10 ml of blood did not impact PCR results in that cohort of chronically infected patients. However, when cumulative results from the combination of the first two samples were computed, higher PCR positivity rates were observed in both cohorts. In cases where samples 1 and 2 were PCR negative, the inclusion of the third sample collected one week later increased sensitivity. Indeed, increasing the number of samples during screening allows enrolling a higher number of patients within the same project period.

In the MSF-DNDi PCR Sampling Optimization Study (Parrado et al., 2019), three samples were taken from all patients regardless of the PCR results in the first two. No statistical differences were observed when testing individual samples. However, when the cumulative PCR positivity of sample 1 plus 2 was compared to the positivity of either of them as a single test, an increase in sensitivity was observed. This was also true when considering the combination of all three samples, resulting in higher PCR positivity rates.

The fluctuation of PCR positivity and non-detectable results can be attributed to the low parasitic burden in chronic Chagas disease patients, which often falls below the limits of detection and quantification of available PCR techniques. In the DNDi-CH-E1224-001 trial, quantitative PCR results showed fluctuations in parasitic loads among positive PCR samples from the same patient, with some samples falling below the limit of quantification. The decision on the optimal opportunity to take an additional sample for PCR testing is currently based on operational and logistical factors rather than in the probability of detecting a particular percentage of additional positives based on the replicative dynamics of the parasite population. This is also applicable to the detection of treatment failure, as conducting more PCR determinations increases the chances of detecting failures.

In reported clinical trials, the measurement of DNAemia at the end of treatment (EOT) regimens was not useful for assessing treatment response. In the DNDi-CH-E1224-001 trial, there was a rapid response in all treatment arms at EOT, but differences in the degree of treatment failure became clear approximately 100 days after treatment initiation. Parasitemia appeared to remain dynamic for up to 6 months after treatment and became more stable afterward. This is likely because some time was needed for refractory or resistant parasite populations to reach a detectable parasitic load threshold in the bloodstream using available PCR procedures.

Different clinical trials have used PCR as an endpoint to evaluate treatment efficacy. In the BENDITA trial, sustained parasitological clearance at six months, defined as persistent negative qualitative PCR results since EOT, was the primary endpoint (Torrico et al., 2018).

The STOP CHAGAS trial evaluated the proportion of subjects with persistent negative PCR by day 180 as the primary efficacy outcome, with negative PCR at 360 days as a secondary outcome (Morillo et al., 2015; Morillo et al., 2017). The EQUITY trial assessed the proportion of enrolled patients testing positive at least once in up to three independent PCR assays, separated by seven days between each, carried out between 12 and 18 months after randomization (Villar et al., 2019).

Currently, clinical trials include PCR measurements at six and twelve months after EOT as common timepoints for post-treatment PCR assessments (**Table 2**).

**Table 2:** PCR timeline points in current clinical trials evaluating regimens of BZN or NFX in chronic CD adult patients.

CLINICAL TRIAL ID	COUNTRY	CLINICAL SETTING	DESIGN	TIMELINE POINTS		TREATMENT					TREATMENT FOLLOW-UP										REFERENCE
				Drug	Regimen	Pretmt	15 d	30 d	60 d	90d	1 m	4 m	6 m	8 m	10 m	12 m	18 m	24 m	30 m	36 m	
**BETTY**	Argentina	Seropositive women with a live birth during pospartum	double-blind, non-inferiority, randomized controlled	BZN	300 mg 60d																Cafferata et al., 2020
					150 mg 30d																
EQUITY*	Colombia	20-65 years old Seropositive without apparent symptoms/ signs cardiomyopathy	randomized concealed blind,, parallel group	NFX	480 mg 60d											3 X					Villar et al., 2019
					240 mg 120d																
				BZN	300 mg 60d																
					150 mg 60d																
				PLACEBO																	
**MULTIBENZ**	Argentina, Brazil Colombia, Spain	Chronic Phase	randomized, noninferiority double blind multicenter	BZN	400 mg 15d																Molina-Morant et al. 2020
					150 mg 60d																
**NUESTROBEN #**	Argentina	18-60 years asymptomatic chronic Chagas disease	phase III, open-label, preospective non-randomized, multicenter, non-inferiority	BZN	300 mg 15d																NCT04897516
					300 mg 30d																
					300 mg 60d																
**TESEO**	Bolivia	Adults chronic phase	open-label, randomized, prospective, phase II	BZN	150 mg 30d																Alonso-Vega et al., 2021
					300 mg 60d																
					150 mg 90d																
				NFX	480 mg 30d																
					480 mg 60d																
					240 mg 90d																

# the dates to perform the PCR controls after EOT varies depending on the duration of each treatment arm

*3X : 3 independent PCR assays separated by 7 days or more between 12 and 18 months of follow-up

Grey boxes represent time line points for PCR analysis.

## Novel molecular techniques potentially useful for monitoring parasitological response to treatment

In recent years, the landscape of DNA amplification and detection techniques has expanded to included highly sensitive isothermal amplification methods such as Loop mediated isothermal amplification (LAMP) and CRISPR-Cas12 technology. Cas12 is a a RNA-guided endonuclease enzyme found in CRISPR-Cas systems, which has been harnessed for its ability to target specific DNA sequences (You et al., 2022). When paired with a guide RNA complementary to the target DNA, Cas12 can be activated upon binding to the target resulting in a collateral cleavage activity that generates detectable signals, such as fluorescence. LAMP employs a set of four to six primers designed to recognize multiple distinct regions on the target DNA enabling a strand-displacement DNA synthesis process. A T.cruzi LAMP prototype kit (Besuschio et al., 2017; Besuschio et al., 2020) has been already tested in blood samples from CD patients under treatment, with promising sensitivity and specificity and high agreement with Real Time PCR used as a comparator test (Muñoz-Calderon et al., 2022).

## Final remarks

In conclusion, the complexity of *T.cruzi* infection, including the coexistence of multiple clones, strains, and discrete typing units in a specific geographic region and patient cohort, as well as the differential histotropism and lack of synchrony among life-stage cycles, poses challenges in predicting an optimal timeline for Real-Time PCR monitoring and assessing the overall parasitological response to treatment. Given these complexities, it is reasonable to maintain PCR tests at baseline, as well as at six and twelve months post-treatment, in order to facilitate the comparison of parasitological responses among different drugs and treatment regimens. This approach allows for a more comprehensive evaluation of treatment efficacy and response in CD clinical trials.

## Author contributions

AS: Conceptualization, Funding acquisition, Writing – original draft, Investigation, Writing – review & editing.
